# Correction: Improving clinical paediatric research and learning from COVID-19: recommendations by the Conect4Children expert advice group

**DOI:** 10.1038/s41390-023-02655-6

**Published:** 2023-06-01

**Authors:** Athimalaipet V. Ramanan, Neena Modi, Saskia N. de Wildt, c4c Learning from COVID-19 Group, Beate Aurich, Sophia Bakhtadze, Francisco J. Bautista Sirvent, Fernando Cabañas, Lisa Campbell, Michaela Casanova, Philippa Charlton, Wallace Crandall, Irmgard Eichler, Laura Fregonese, Daniel B. Hawcutt, Pablo Iveli, Thomas Jaki, Bosanka Jocic-Jakubi, Mats Johnson, Florentina Kaguelidou, Bülent Karadag, Lauren E. Kelly, Ming Lim, Carmen Moreno, Eva Neumann, Cécile Ollivier, Mehdi Oualha, Genny Raffaeli, Maria A. Ribeiro, Emmanuel Roilides, Teresa de Rojas, Alba Rubio San Simón, Nicolino Ruperto, Maurizio Scarpa, Matthias Schwab, Angeliki Siapkara, Yogen Singh, Anne Smits, Pasquale Striano, Silvana A. M. Urru, Marina Vivarelli, Zorica Zivkoviz

**Affiliations:** 1 University Hospitals Bristol NHS Foundation Trust, Bristol, UK; 2 Translational Health Sciences, University of Bristol, Bristol, UK; 3 Section of Neonatal Medicine, Department of Public Health and Primary Care, Imperial College London, London, UK; 4 Chelsea and Westminster NHS Foundation Trust, London, UK; 5 Department of Pharmacology and Toxicology, Radboud Institute for Health Sciences, Radboudumc, Nijmegen, The Netherlands; 6 Intensive Care and Department of Paediatric Surgery, Erasmus MC Sophia Children’s Hospital, Rotterdam, The Netherlands; 7 Robert Debré University Hospital, Paris, France; 8 Tbilisi State Medical University, Tbilisi, Georgia; 9 Hospital Niño Jesús, Madrid, Spain; 10 Quironsalud Madrid University Hospital, Madrid, Spain; 11 Biomedical Research Foundation, La Paz University Hospital-IDIPAZ, Madrid, Spain; 12 Medicines and Healthcare Products Regulatory Agency MHRA, London, UK; 13 Fondazione IRCCS Istituto Nazionale dei Tumori di Milano, Milan, Italy; 14 UCB Biopharma, Anderlecht, Belgium; 15 Eli Lilly and Company, Indianapolis, IN USA; 16 European Medicine Agency EMA, Amsterdam, The Netherlands; 17 Department of Women’s and Children’s Health, University of Liverpool, Liverpool, UK; 18 NIHR Alder Hey Clinical Research Facility, Alder Hey Children’s Hospital, Liverpool, UK; 19 Bayer Aktiengesellschaft, Barmen, Germany; 20 Department of Mathematics and Statistics, Lancaster University, Lancaster, UK; 21 MRC Biostatistics Unit, University of Cambridge, Cambridge, UK; 22 Clinical Center Nis, Pediatric Clinic, Child Neurology Department, Belgrade, Serbia; 23 Sultan Qaboos University Hospital, Department of Pediatric Neurology, Muscat, Oman; 24 Gillberg Neuropsychiatry Centre, Sahlgrenska Academy, Gothenburg University, Gothenburg, Sweden; 25 Clinical Investigations Center, CIC1426, Robert Debré Hospital, Assistance Publique des Hôpitaux de Paris (APHP), Paris, France; 26 Université de Paris, Paris, France; 27 Marmara University Faculty of Medicine, Istanbul, Turkey; 28 Department of Pediatrics and Child Health, University of Manitoba, Children’s Hospital Research Institute of Manitoba, Winnipeg, MB Canada; 29 Evelina London Children’s Hospital, Guys and St Thomas’ NHS Foundation trust, London, UK; 30 Department Women and Children’s Health, Faculty of Life Sciences and Medicine, King’s College London, London, UK; 31 Department of Child and Adolescent Psychiatry, Institute of Psychiatry and Mental Health, Hospital General Universitario Gregorio Marañón, School of Medicine, Universidad Complutense, IiSGM, CIBERSAM, Madrid, Spain; 32 Dr. Margarete Fischer-Bosch-Institute of Clinical Pharmacology, Stuttgart, Germany; 33 Aparito, Wrexham, UK; 34 EA7323, University of Paris, Paris, France; 35 Pediatric Intensive Care Unit, AP-HP, Necker Hospital, Paris, France; 36 Department of Clinical Sciences and Community Health, University of Milan, Milan, Italy; 37 NOVA Medical School, Faculdade de Ciências Médicas, Universidade Nova de Lisboa, Lisboa, Portugal; 38 Aristotle University of Thessaloniki, Thessaloniki, Greece; 39 Hippokration Hospital, Athens, Greece; 40 Children’s University Hospital Niño Jesús, Madrid, Spain; 41 IRCCS Istituto Giannina Gaslini, Genova, Italy; 42 Regional Coordinating Center for Rare Diseases, University Hospital, Udine, Italy; 43 Department of Clinical Pharmacology, University Hospital, Tübingen, Germany; 44 Cambridge University Hospitals NHS Foundation Trust, Cambridge, UK; 45 University of Cambridge School of Clinical Medicine, Cambridge, UK; 46 Neonatal intensive care unit, University Hospitals Leuven, Leuven, Belgium; 47 Department of Development and Regeneration, KU Leuven, Leuven, Belgium; 48 University of Genova, Genova, Italy; 49 Chiara Hospital Trento, Trento, Italy; 50 Division of Nephrology and Dialysis, Department of Pediatric Subspecialties, Bambino Gesù Pediatric Hospital IRCCS, Rome, Italy; 51 Children’s Hospital for Lung Diseases & Tb Clinical Center Dr Dragiša Mišovic, Belgrade, Serbia; 52 Faculty of Pharmacy Novi Sad, Business Academy, Novi Sad, Serbia

Correction to: *Pediatric Research* (2021) 91:1069–1077; 10.1038/s41390-021-01587-3

The following publication disclaimer was added to the article:

“The publication reflects the author’s view and neither IMI nor the European Union, EFPIA, or any Associated Partners are responsible for any use that may be made of the information contained therein.”

The original article has been corrected.

